# OA32 Minimally invasive, ultrasound-guided tissue biopsies of synovial tissue in children with Juvenile Idiopathic Arthritis for research: a feasibility study

**DOI:** 10.1093/rap/rkac066.032

**Published:** 2022-09-28

**Authors:** C Bolton, C G Smith, A McNeece, S Sultan, V Alexiou, A Hackland, J Crook, H D Nguyen, Cluster Champions, M Thyagarajan, Z Shiekh, C Cotter, P Reis Nisa, E Al-Abadi, S Chippington, S Compeyrot-Lacassagne, A Filer, L Wedderburn, Adam Croft

**Affiliations:** UCL GOS ICH, London, United Kingdom; NIHR BRC at GOSH, London, United Kingdom; University of Birmingham, Birmingham, United Kingdom; UCL GOS ICH, London, United Kingdom; Birmingham Women’s and Children’s NHS Foundation Trust, Birmingham, United Kingdom; UCL GOS ICH, London, United Kingdom; NIHR BRC at GOSH, London, United Kingdom; University of Birmingham, Birmingham, United Kingdom; UCL GOS ICH, London, United Kingdom; UCL GOS ICH, London, United Kingdom; CLUSTER Consortium, UK wide, United Kingdom; Birmingham Women’s and Children’s NHS Foundation Trust, Birmingham, United Kingdom; Birmingham Women’s and Children’s NHS Foundation Trust, Birmingham, United Kingdom; University of Birmingham, Birmingham, United Kingdom; University of Birmingham, Birmingham, United Kingdom; Birmingham Women’s and Children’s NHS Foundation Trust, Birmingham, United Kingdom; Great Ormond Street Hospital, London, United Kingdom; NIHR BRC at GOSH, London, United Kingdom; Great Ormond Street Hospital, London, United Kingdom; NIHR BRC at GOSH, London, United Kingdom; University of Birmingham, Birmingham, United Kingdom; UCL GOS ICH, London, United Kingdom; Great Ormond Street Hospital, London, United Kingdom; NIHR BRC at GOSH, London, United Kingdom; University of Birmingham, Birmingham, United Kingdom; NIHR BRC at Birmingham, Birmingham, United Kingdom

## Abstract

**Introduction/Background:**

When investigating disease mechanisms, site-specific differences in immune cell phenotype and function have highlighted the need to analyse cellular and molecular mechanisms at the tissue site directly. In adults, the ability to obtain synovial tissue biopsies using ultrasound-guided techniques, combined with advanced tissue analytics, has revolutionised our understanding of the cellular ecosystem that operates within the joint and how it contributes to disease. However, a similar approach in paediatric disease is lacking.

**Description/Method:**

Aims: 1) To describe the protocol for undertaking minimally invasive ultrasound-guided synovial tissue biopsies in children and young people with arthritis, for the purpose of research, alongside routine clinical care. 2) To investigate whether high-quality synovial tissue can be obtained that is suitable for downstream applications including single cell profiling technologies, histology and digital spatial profiling.

Treatment-naïve children with a diagnosis of Juvenile Idiopathic Arthritis, who were being referred for a corticosteroid joint injection were recruited from two large UK Paediatric Rheumatology centres. We established a workflow pipeline for performing synovial tissue biopsies in child and young people with arthritis, using standardised procedures for biopsy and sample processing. Procedures were performed by experienced paediatric interventional radiologists with experience of joint biopsy for diagnostic purposes. Following a general anaesthetic, required as part of routine clinical care and the establishment of sterility, synovial fluid was aspirated. Needle-biopsies were undertaken from the same needle insertion site and subsequently corticosteroid was injected into the joint. Thickened synovium was graded via ultrasonography. Participating families completed questionnaires prior to and following synovial biopsy.

**Discussion/Results:**

11 participants were recruited to the study over a nine month period, with a median age of 7 years (range 1-16 years); 91% were female. Samples obtained included core synovial biopsies, paired synovial fluid and peripheral blood. Synovial tissue fragments were processed for histology by formalin fixation and cryopreserved for downstream applications, including RNA sequencing and cell culture. Quality control indices included histological analysis to ensure the biopsied material was characteristically synovium and to grade the severity of inflammation. No significant complications were reported; however, one child had a mild haemarthrosis controlled with cold saline wash out and cold compresses.

**Key learning points/Conclusion:**

Obtaining biopsies of synovial tissue in children with Juvenile Idiopathic Arthritis for the purpose of research, alongside clinical care is feasible. Analysis of tissue direct from the site of inflammation with single-cell RNA sequencing in children is achievable.

